# Qualitative study of psychosocial factors impacting on Aboriginal women’s management of chronic disease

**DOI:** 10.1186/s12939-019-1110-3

**Published:** 2020-01-13

**Authors:** A. Eades, M. L. Hackett, H. Liu, A. Brown, J. Coffin, A. Cass

**Affiliations:** 1 The George Institute for Global Health, The University of New South Wales, PO Box M201, Missenden Road, Sydney, NSW 2050 Australia; 2 The University of Sydney, Sydney, Australia; 3 South Australian Health & Medical Research Institute, Adelaide, South Australia; 4 Telethon Kids Institute Australian Medical Research Institute, Perth, Western Australia; 5 Menzies School of Health Research, Charles Darwin University, Darwin, Northern Territory Australia

**Keywords:** Aboriginal women, Chronic disease, Health, Social determinants of health, Intergenerational, Incarceration, Mental health, Domestic violence, Cultural security, Flexible model of service delivery

## Abstract

**Background:**

Aboriginal women are frequently called upon to support their families and other community members. At times, such supporting roles can be burdensome for these women. Many Aboriginal women live with chronic conditions. We explored the ways in which the women’s caring roles impacted on how they maintained their own health.

**Methods:**

The aim of this manuscript is to explore the psychosocial factors associated with the management of health and chronic disease in Aboriginal women. An interpretive phenomenological approach was used for the analysis of 72 in-depth semi-structured interviews. These interviews were conducted in four community controlled Aboriginal health services, in urban, rural and remote settings, across two states and a territory in Australia.

**Results:**

Women living with chronic disease experience multiple challenges while caring for family, such as intergenerational trauma, mental health issues relating to addiction, domestic and family violence and incarceration. When these women become ill, they also have to take care of themselves. These women provided informal and unfunded care in response to a range of complex family and community problems. This continuous caring for family affected the women’s ability to maintain their health and manage their own chronic conditions.

**Conclusion:**

The caring roles and responsibilities Aboriginal women have in their community impact on their health. Aboriginal women provide much needed refuge and support to family and the wider community. Underfunded and over-burdened formal support services are not meeting the needs of many Aboriginal women. Improved culturally secure resources and social services are required within communities to support Aboriginal women to successfully manage their own health.

## The known

Aboriginal women’s health is improving, however inequalities persist.

## The new

Women take on complex roles and responsibilities within their families and communities. With insufficient access to culturally appropriate social support services, women describe negative impacts on their health.

## The implications

Improved access to culturally secure social services is essential to improve the physical and mental health of Aboriginal women.

## Background

Australian Aboriginal and Torres Strait Islander peoples (from here on referred to as Aboriginal peoples), are among the oldest continuous living cultures in the world, with approximately 260 language groups that co-existed prior to colonization, each with their own customs and practices [1]. Yet the social determinants of health for Aboriginal people are significantly worse leading to poorer health outcomes compared to other Australians. Current estimates of the Life Expectancy (LE) gap for Aboriginal people are 8.6 (urban) and 13.8 years (rural and remote) for Aboriginal men, and 7.8 (urban) and 14 years (rural and remote) for Aboriginal women [2, 3]. Yet for many Aboriginal women, it is not uncommon to have key responsibilities in caring for many others in their families as well as caring for their own health.

Chronic disease is the leading cause of death for Aboriginal people aged between 35 and 74 years [4]. Premature mortality due to cardiovascular disease (CVD), diabetes and chronic kidney disease (CKD) contributes substantially to the life expectancy gap [5]. In 2012–13, 38% of the total adult Aboriginal population had two or more chronic conditions relating to Diabetes, CVD or CKD. This proportion represents a 12% absolute difference compared to 26% of the non-Aboriginal population [5]. Reduced access to preventive health services, patterns of risk-taking behavior and socioeconomic disadvantage contribute to these health inequalities [5, 6].

Aboriginal women have experienced intergenerational trauma related to current and past policies of dispossession, infant and child removal, dislocation from lands, incarceration, and homelessness which has placed many Aboriginal women and their families at risk of disadvantage [7]. These experiences are reported in the *2015 Close the Gap Priorities and Progress Report on social determinants of health* which reiterated the link between socioeconomic disadvantage and poor health outcomes [8, 9]. Late presentation for medical care by many Aboriginal women is one factor impacting on Aboriginal women’s health [10]. There are many reasons for this, including key roles the women have within their communities. These roles extend far beyond the structure of the nuclear family, as do the expectations placed on the women [11]. Aboriginal women take care of the sick in their household potentially at the expense of their own health [11, 12].

We aimed to explore whether the roles women have in their family impact on them caring for themselves. We know that conflicting demands such as caring for a sibling’s children while one parent is in prison, caring for family affected by mental ill health or caring for extended families’ infants to prevent them from entering the child protection system contribute to the reported high levels of stress for many Aboriginal women [13]. A 2008 National Indigenous Social Survey conducted by the Australian Bureau of Statistics (ABS) reported that 80% of Aboriginal people who participated in the survey had experienced at least one significant stressor in the year before participating in the survey [14]. The impact of these stressors on chronic disease management for Aboriginal women is poorly understood.

## Methods

The findings reported here are presented according to Consolidated Criteria for Reporting Qualitative Research (COREQ) [15].

### Study design and participants

A purposive sampling approach was used to select participants for this study. Women were eligible if they identified as Aboriginal and/or Torres Strait Islander; had been diagnosed with diabetes, CKD or CVD; and were attending one of four urban, rural or remote Aboriginal Medical Service (AMSs) in two states and one territory in Australia between May and October 2014.

### Procedures

A clinical staff member at each AMS assisted AE to recruit participants, AE conducted one-on-one semi-structured interviews with consenting participants following an interview guide (see Interview Guide Additional file 1) at the local AMS or in their homes. Interviews lasted between 15 and 60 min. All 72 interviews were audio-recorded. At the completion of interviews, demographic data were collected. Interviews were transcribed and de-identified by an independent transcription service. Due to time constraints at each AMS, interviews were conducted in blocks and transcription occurred after all interviews were completed.

### Analysis

Interpretive Phenomenological Analysis (IPA) [14, 15] was used to identify patterns of meaning. This approach was used as it is concerned with exploring and understanding the lived experiences of participants by detailing their world view and the meaning they attached to those experiences [16]. Coding was conducted independently by AE and HL using NVivo 10. Having reviewed five transcripts, AE and HL met to discuss emerging themes, and combine codes into common themes (see Code Book Additional file 2). AE and HL then met with the research team to discuss the emerging themes and coding framework. Subsequent meetings of the research team, at stages throughout analysis of the 72 interviews, reviewed and further discussed the coding framework, including in relation to new emerging themes.

### Research team and reflexivity

AE is an Aboriginal woman, registered nurse with a Doctor of Philosophy. AE had an existing professional or personal relationship with each of the AMSs before the commencement of any negotiations, consultations or agreement to conduct research at their service. HL, medically trained with a PhD, assisted with the coding; AB and JC are both leading Aboriginal researchers and AB is also a medically trained doctor. MH, HL and AC are non-Aboriginal researchers with medical and/or epidemiology backgrounds who conduct research in collaboration with AMSs and Aboriginal people.

### Ethics approval

This study was approved by the Far North Queensland Human Research Ethics Committee (reference HREC/13/QCH/118–870), Aboriginal Health Council of Western Australia (reference HREC Reference Number 535) and the Central Australian Human Research Ethics Committee (Reference Number HREC-14-212).

## Results

Seventy-two Aboriginal women were interviewed from Western Australia, Queensland and Central Australia. They ranged in age from 26 to 80 years with a mean age of 53 years. All the women reported managing diabetes, while some of the women also managed chronic kidney disease or cardiovascular disease, and a few managed all these conditions combined. No one reported depression or anxiety.

The women spoke of many stressors that impacted on their ability to manage their chronic disease. These stressors related to caring for family and community, and supporting other family households as well as their own. The women spoke of intergenerational trauma, drug and alcohol misuse, domestic violence and incarceration and how these contributed to the breakdown of family support networks. Women discussed the support they had access to, including formal services and informal family and community support. Overall the stressors and supports were grouped into the following interrelated domains: stressors affecting the women, their families and community; support from families; and lack of care for the carers.

### Stressors affecting the women, their families and community: competing roles and responsibilities, death and dying, and intergenerational trauma as a burden

The women described different family structures including nuclear families, one parent families and extended families, however all described having significant caring responsibilities. This included supporting family when they came into town to see specialist doctors and having to care for additional family members in the household for reasons such as death, illness, child removal, homelessness and incarceration. There was also regular attendance at funerals and associated sorry business. Other significant caring responsibilities came about following family violence, intergenerational historical and present-day trauma, and family members experiencing drug and alcohol related illness before seeking medical care. This was very common when mental health services were needed for family members.

Caring was described by the participants as a role and a responsibility. Women expressed feeling worn down and not being able to manage their own health conditions because of their caring roles. Caring frequently extended beyond their own children and included grandchildren and other members of their extended family.

*“I’ve had them while looking after [my brother’s] daughter’s kids, because he was in prison; the five of them. Plus my daughter’s five, plus my other daughter’s three, 13 all at once …then I got sick and had to make the hard decision to hand the kids back into care – it took me until I got very sick to realise that I couldn’t do it anymore.” (Participant 02, Urban AMS)*

*“I still can’t get my diabetes down under 10, it’s a struggle. It’s got a lot to do with your everyday living, it’s not a scheduled white non-Indigenous way. You wake up and you’re traumatized, before the day has ended. They’ve (my kids) damaged the house, you know. He’s (my husband) forever patching the walls, because they’ve punched it, kicked it, smashed globes; kicked the gate in and kicked the garage door in. You forget your insulin, you forget your medication or you’re taking it too late.” (Participant 11, Urban AMS)*

*“I’ve been babysitting my granddaughter all the time, I told my daughter, you need to give me a break so that I can concentrate on taking my medications. When I’m with her, I can’t be responsible for myself, I find it hard.” (Participant 11, Rural AMS).*

Death and dying were common occurrences. End of life care, death and sorry business were intricately woven into the daily lives of Aboriginal families and communities. A death in a family was the loss of a family member, a community member and a client of the health service. It was a communal experience.

*“There was 18 months, we had all this grief…I got really depressed with all the deaths. It’s not only the families, it’s the community that’s constantly grieving.” (Participant 19, Regional AMS).*

Some women described how people became overwhelmed by grief after the loss of close family and community members.

*“Reckon we grieve too heavy, that’s how our culture is,… grieving, and some people haven't been taught how to pull themselves out of that , we grieve far too heavily where people just become totally depressed.” (Participant 19, Regional AMS).*

A lack of flexibility and cultural understanding from others about the sorry business was keenly felt by those in paid work.

*“In the last year I’ve lost five family members from bush. And … working in town and having time to go back just for the funerals is impossible. We get 10 days bereavement. To go back for a bush funeral is a couple of weeks. So, …I can go out on the day they’re getting buried and come back the same day, you know, …, that’s hard because when my two older sisters, … passed away, my family out bush doesn’t understand all the paperwork [I have to provide to my workplace].” (Participant 13, Remote AMS).*

The high and intergenerational incarceration rates and rates of institutionalisation across the communities had an impact well beyond the individuals directly affected.

*“…my second eldest son is pretty stressed out. (He) is in jail for 10 years. His father has been in and out of prison since the day my son was born.” (Participant 10, Regional AMS Site).*

*“I worry about my granddaughter, she’s got three little children, (and) she’s only in her twenties and her boyfriend, he’s in prison.” (Participant 20, Regional AMS Site).*

Another woman spoke of the impact of drugs and alcohol on her family which resulted in her son being incarcerated, first in prison and then, because he had mental health issues, in a mental health facility.

*“My oldest boy, he’s in… [a] mental health [hospital]…he’s been there for the last five years.” (Participant 2, Remote AMS Site).*

Drugs and alcohol were described by all participants as a fueling factor in intimate violence.

*“They were perfect good grandparents and good parents, unless they drank, you know. And then that drink…it takes our heroes and makes them into evil pigs, and the men have got to know that, you know, they’re the warriors, they have to stand up and start protecting us, not abusing us, not being our enemy.” (Participant 12, Regional AMS)*

*“I met my first partner – I mean he was a good person but he just did drugs and I couldn’t handle drugs and alcohol – I guess because I saw my mother drinking all the time and I didn’t want to get in that predicament of being an alcoholic. ” (Participant 2, Regional AMS)*

*“He hit me in the back of the head with an empty beer bottle because I wouldn’t stop to get him another one out of the boot of the car, the roadhouse was only a mile away.” (Participant 18, Urban AMS).*

### Support: lack of formal support, family as a strength, cultural identity and country

Formal support services such as police, women’s shelters, legal services, hospitals and community services were not used as often as needed because these services were not considered culturally secure or safe. Participants reported feeling embarrassed to use these services.

“*It’s really poor … because … I’ve seen women that I think, don’t have the knowledge of what’s out there for them to access. You know, there is no place where they can go and say that they are sick. Maybe its cultural reasons, [because] for women’s health and dealing with pap smears, and breast you know, all that sort of stuff is embarrassing. Also, the old people, my mum for instance, never used to do all of that stuff [getting women’s health checks]… it’s (seen as) a sacred thing.”. (Participant 18, Regional AMS)*

Another woman shared how although there is a women’s health service, there is no female GP at the health service. She said:

*“… As for services for women’s health, [name of health service] do not have a lady doctor here at the moment in this area, in the generalist clinic. They haven’t had one here for a while now, it’s a bit hard if you want to see a lady doctor” (Participant 1, Regional AMS).*

For these Aboriginal women, family values were very important. The extended nature of many Aboriginal families means that the women’s roles and responsibilities were broad, and the expectations placed on them to care for others, were great. While the grandmother, mother, sister and aunty play vital roles in maintaining stability within the family, this also places additional stress on them. The women considered family as being their main source of support – especially in environments where ‘the system’ did not provide adequate assistance.

*“Well, I don’t worry about them too much [my kids and grandchildren]. Financially, it’s just that they don’t have much, and they know how to draw on my purse strings, [for example], if we’ve got a show on that’s coming to school and we don’t have any money to pay for the show. Or, they [the grandchildren], need uniforms or medications – I now have an account at the chemist so they just go and add on my account” (Participant 3, Regional AMS Site).*

The women identified education as being important to gain employment, leading to good health and wellbeing outcomes, and their ability to lead a better life. They often supported other family members while they undertook formal study.

“*My son-in-law can’t do any work because she [daughter] can’t handle the two kids. One of her kids has autism and she is flat out looking after things, and she is doing her studies, and you know, lucky I am a strong person and I don’t get upset easily or, you know, I go and talk to them the best that I can and look after them.” (Participant 20, Regional AMS)*

*“My daughter used to go and work, or doing studies and while they are away, they let me look after the grandchildren.” (Participant 20, Regional AMS)*

Another woman shared how as a family, they took turns in caring for her sister during her sister’s ordeal with breast cancer. She said:

*“My sister had breast cancer a couple of years ago, we looked after her for that time. We took it in turns to visit her every day, cook meals for her during the week because she wasn’t eating well. But she’s come through that now”* (Participant 1, Regional AMS Site).

It was not uncommon for women working in the health system to describe coordinating the care for the family, and during their lunch hour, going home and checking on elderly parents or siblings to make sure they had eaten lunch.

“*Mum has meals on wheels set up for her now. But I used to just go to work – come to work, pick him [dad] up at lunch time*, *drop him at my place so that we could eat together that night and then take him home to his place – that used to be the routine. [He] pretty much got a room now - it will probably come to the point where we [me, mum and dad] will all be under the same roof. Because [the running between places] it’s just starting to get too much for me”.* (Participant 6, Regional AMS Site)

Yet the women, when reflecting on some of their and their family’s experiences, also shared stories describing great strength, leadership, strong family and breaking the cycle of intergenerational trauma. For example, one woman described her lived experience over three generations;

*“Nanna had eight kids by grandfather, for one of her babies, (he) kicked her until the baby was dead and she was a mess.*

*“… My parents had horrific … childhoods of starvation, of abuse, sexually, abuse by the nuns, the priest… my mum’s Methodist church minister raped her. You know, the whole of my parent’s childhood was ugly.*

*“…I had a beautiful childhood. Dad taught us how to track and told us stories, and we had campfires and he built cubbies and everything…we were poor, but we didn’t go without too much.” (Participant 12, Urban AMS).*

The strength and resilience in this family is evident by the woman’s ability to tell her story, and by the strength of her father and the women who came before her.

### Lack of care for the carers

The women took great pride in speaking about their caring roles, and how they cared for so many people in their families. However, there did not seem to be any expectation of being cared for by others when these women became ill. When asked who cared for them when they were sick few of the women named or suggested others providing formal or informal care for them. This information was only provided when questioned directly.

*“I can’t afford to get sick. If I’m sick I still need to complete my role that I always do. I just grin and bear it. Before when the children were small, (even if I was sick), I still used to get out of bed and take them to school.” (Participant 16, Remote AMS)*

*“If I get sick this way, I make sure I don’t panic, then if it gets worse, I just ring the ambulance then go to hospital.” (Participant 14, Remote AMS)*

*“I look after myself.” (Participant 2, Remote AMS)*

The reluctance with which the participants answered this question and the fact that none of them dwelled on it, seemed to indicate they did not prioritise caring for their own health above others.

## Discussion

With this research we aimed to understand the roles and responsibilities taken on by Aboriginal women within their families and communities and how this affected their ability to manage their own health and chronic conditions. Although all the women had at least one chronic disease, very little of their conversation focused on their illness. The women spoke of the stressors affecting them, and their families. They talked about their competing roles and responsibilities, intergenerational trauma, death and dying, and shared how caring was not an easy task; in fact, it could become very burdensome. Yet, it is a role that is normalized and expected as an Aboriginal woman within a family unit. They saw this role as essential and provided care to others, at times, at the expense of their own health.

The formal support systems such as the police and women’s shelters were not used as often as needed because these services were seen as not being accessible or culturally secure for Aboriginal women. Instead, the women were providing services that should have been provided by drug and alcohol, mental health, domestic and family violence and homelessness service providers. These complex roles that the women were responsible for within their families, and the duration of these roles and responsibilities that often go unreported, were substantial. These burdens are a result of intergenerational trauma stemming from Aboriginal children being removed from their families, a practice which continues in the present day. The ongoing experiences of racism, poverty and disproportionate incarceration represent additional burdens for Aboriginal people in Australia [17–20]. Therefore, rather than use the formal services, they used the informal support networks within their own family, extended family and community. These informal supports had the required cultural integrity and the women were able to use them while remaining on their own country.

Cultural safety, which recognises Indigenous knowledge, beliefs and values, is a significant factor for Aboriginal people in accessing available services [21]. Davy et al. (2016) identified that while Aboriginal people with acute health issues recognised the importance of managing their health, they were unlikely to access available services if those services were not culturally welcoming or inspiring a sense of trust [22]. Durey & Thompson (2012) reiterate the importance of cultural safety in health settings for Aboriginal people. Their study found that improved health for Aboriginal people requires a shift in policy and practice away from standardised care to one that incorporates Indigenous knowledge, values and beliefs. As they concluded, such a shift is required to ensure that health services, not Aboriginal clients themselves, are asked to step up to provide culturally secure and high-quality care [21].

The health of Aboriginal women with chronic disease who have multiple caring roles is at serious risk due to the lack of culturally appropriate health and social services. When directly asked ‘who looks after you’ these women did not readily identify other people who cared for them. They talked about being responsible for their own care. Monahan et al. (2007) highlighted in their carers’ counselling guidelines that the lack of culturally appropriate services are a barrier to access by Aboriginal people [23]. While similar to the current study, Hill et al. (2012), described a direct link between the informal caring that occurs amongst Indigenous people, and increased rates of disability, chronic disease, and long term health conditions [24].

### Strengths and limitation

The strengths of this paper are that we have been able to provide insights into lived experiences and the extent to which psychosocial factors impact the management of chronic disease for the women in this study. A limitation of this study is that we sought to include women living with a chronic disease which led to us recruiting few younger and healthier women whose views may differ from the views reported here.

## Conclusion

The women in this study seldom spoke of themselves or of their chronic health conditions, unless directly prompted to do so. They had an outward focus towards supporting the needs of their family and community, highlighting the many roles they were culturally responsible for fulfilling, sometimes at the expense of their own health. Yet, because health and social services were not culturally secure, the women were not using available formal support services in their community. Therefore, the health of Aboriginal women with chronic disease is affected due to a lack of culturally secure services, leaving women juggling multiple caring roles and responsibilities, while potentially neglecting their own health.

## Supplementary information

**Additional file 1.** Interview guide.

**Additional file 2.** Code book

**Additional file 3.** Node structure

## Data Availability

The data has been de-identified and is being stored in an NVivo 10 Database which is stored on a password protected computer. All hard copies of consent forms and identifiable data is stored at the lead researcher’s organisation in a locked filing cabinet in a lockable office.

## Glossary

Aboriginal or Torres Strait Islander: a person of Aboriginal and/or Torres Strait Islander descent who identifies as an Aboriginal and/or Torres Strait Islander.
Burden of disease: term referring to the quantified impact of a disease or injury on an individual or population. It is measured using the disability-adjusted life year, which is a year of healthy life lost through either premature death or living with disability due to injury or illness.
Cardiovascular disease (CVD): Diseases affecting the circulatory system, namely the heart (cardio) or blood vessels (vascular). Includes heart attack, angina, stroke and peripheral vascular disease. CVD is also known as circulatory disease.
Chronic diseases: term applied to a diverse group of diseases, such as heart disease, cancer and arthritis, which tend to be long lasting and persistent in their symptoms or development. Although these features also apply to some communicable diseases, the term is usually confined to non-communicable diseases.
Chronic kidney disease: Relates to all the conditions of the kidney, lasting at least 3 months, where a person has had evidence of kidney damage and/or reduced kidney function, regardless of the specific diagnosis of disease or condition causing the disease.
Comorbidity: When a person has two or more health problems at the same time.
Cultural Security or (culturally secure): Is a commitment to the principle that the provision of services offered by a system or service will not compromise the legitimate cultural rights, values and expectations of Aboriginal and Torres Strait Islander people. Cultural security refers to embedded structures and policies which aim to ensure Aboriginal people feel safe in accessing health or social services,
Diabetes (diabetes mellitus): A chronic condition in which the body cannot properly use its main energy source, the sugar glucose. This is due to either the pancreas not producing enough of the hormone insulin or the body being unable to effectively use the insulin produced. Insulin helps glucose enter the body’s cells from the bloodstream and then be processed by them. Diabetes is marked by an abnormal build-up of glucose in the blood and it can have serious short-term and long-term effects on any of the body’s systems, especially the blood vessels and nerves.
Dialysis: an artificial method of removing waste products and water from the blood as well as regulating levels of circulating chemicals. There are two main forms of dialysis: haemodialysis (which occurs outside the body via a machine) and peritoneal dialysis (which occurs inside the patient’s body via the lining of the abdominal cavity).
End-stage kidney disease: the most severe form of chronic kidney disease, also known as Stage 5 chronic kidney disease or kidney failure. People with end-stage kidney disease generally experience a range of symptoms and abnormalities in several organ systems due to severe loss of kidney function. Kidney replacement therapy in the form of dialysis or a kidney transplant is required for survival when kidney function is no longer sufficient to sustain life.
High blood pressure/hypertension: The definition of high blood pressure (also known as hypertension) can vary, but a well-accepted one is from the World Health Organization: a systolic blood pressure of 140 mmHg or more or a diastolic blood pressure of 90 mmHg or more, or [the person is] receiving medication for high blood pressure.
Risk factor: Any factor which represents a greater risk of a health disorder or other unwanted condition or event. Some risk factors are regarded as causes of disease, others are not necessarily so. Along with their opposites—protective factors—risk factors are known as determinants.
