# Supplementary material for: Qualitative study of psychosocial factors impacting on Aboriginal women’s management of chronic disease
Source: Int J Equity Health. 2020 Jan 13;19:8. doi: 10.1186/s12939-019-1110-3 (PMC6958573; doi:10.1186/s12939-019-1110-3)
Supplement: Supplementary file 1 — Additional file 1. Interview guide. [file 12939_2019_1110_MOESM1_ESM.pdf]

Questions to Participant

Demographics

1. Can you tell me a bit about where you come from?

Roles in Household/Community

2. What about your family – can you tell me how many people are in your family and how they are related to you?

   Do you all live together? How many people live with you in your house?

3. We would like to better understand all of the things that you do for this family.

   - Can you give me an idea of some of the things that you do?

   - What are your roles in looking after the family?

4. Can you tell me who takes care of the house for things like cleaning, shopping, paying the bills, cooking and getting the kids off to school?

5. What about in your community – can you tell me what roles or jobs you have in your community?

Health

6. When someone in your family gets sick and can't look after themselves, who takes care of them at home?

7. What happens when you get sick? Does anyone look after you? Can you tell me?

8. Think about your health problems, can you tell me what happens when you get sick?

9. Since being diagnosed with the chronic disease, has there been any impact or changes on how you take care of your family or get your work done?

10. What happens to your jobs in the community if you get sick?

11. Can you tell me how you feel or what you think - when people ask you about Aboriginal women's health and wellbeing?-

<u>Social and Emotional Wellbeing</u>

12. Do you worry about your family

13. Can you tell me about that

14. Does it affect how you look after your own health?

15. Always having to worry that your family are alright – how does this make you feel?

16. Do you ever feel as though it's all too much?

17. Where do you get this help from?
