# Supplementary material for: Qualitative study of psychosocial factors impacting on Aboriginal women’s management of chronic disease
Source: Int J Equity Health. 2020 Jan 13;19:8. doi: 10.1186/s12939-019-1110-3 (PMC6958573; doi:10.1186/s12939-019-1110-3)
Supplement: Supplementary file 2 — Additional file 2. Code book [file 12939_2019_1110_MOESM2_ESM.pdf]

Qualitative Interviews – Aboriginal and Torres Strait Islander Women.

Nodes

Name	Description
STRESSORS Affecting the women, their family and community	The stressors affecting the women, their families and community included the competing roles and responsibilities they had to their family and community. Death and dying was also an extreme stressor for the women, intergenerational trauma was described as impacting negatively on the women. Perhaps the most important stressor for the participants was the burden of reciprocity in relation to caring for family.
Roles and responsibilities	The duties that the women identified as being important for them to fulfil as part of their duties to their family and community. The burden existed when the women were unable to fulfil the duties because of lack of capacity.
Death and dying	A significant stressor which most of the women in this study experienced in their family and/or community
Intergenerational Trauma	The emotional and physical trauma experiences carried from grandparent, to parent to sibling
Burden of reciprocity	Feeling as though you have to carry out duties because these duties are seen as part of your responsibilities
Intergenerational Circumstances	Intergenerational trauma related to infant and child removal which included grandparents, parents and participants all being removed from their parents; parents, the women and their children all experiencing domestic violence, the experiences of death and dying and the accumulation of trauma being passed through generations.
Roles and responsibilities	The duties that the women identified as being important for them to fulfil as part of their duties to their family and community
Supports	These are the networks that supported the women and their families.
Formal Support	These were services such as police, women's shelters, legal services, hospitals and community services departments.

Name	Description
Informal Support	Informal supports where family fulfilling the roles and responsibilities and circumstances where family was a strength.
Informal Support through education	The women reported that getting an education enabled them to improve the outlook for them and their family
Informal support through education which led to encouragement to improve health	The women also reported that the education they received supported them to get good jobs and where then able to improve their living circumstances.
Informal support - financial benefits to family	Family financially supported other family members in need so that they did not have to do without food or medications
Identity and country	Identity and country is important for the younger generations. These younger generations will need their elders to pass on all of the knowledge to them.
