# Supplementary material for: Qualitative study of psychosocial factors impacting on Aboriginal women’s management of chronic disease
Source: Int J Equity Health. 2020 Jan 13;19:8. doi: 10.1186/s12939-019-1110-3 (PMC6958573; doi:10.1186/s12939-019-1110-3)
Supplement: Supplementary file 3 — Additional file 3. Node structure [file 12939_2019_1110_MOESM3_ESM.pdf]

Participant Consent Form

I ,.. [PRINT NAME], give consent to my participation in the research project:

Exploring the impact of Chronic Disease on the roles and responsibilities of Aboriginal and Torres Strait Islander women: Is there a relationship between stress, psychosocial health and management of chronic disease for Aboriginal women?

In giving my consent I acknowledge that:

1. The procedures required for the project and the time involved have been explained to me, and any questions I have about the project have been answered to my satisfaction.

2. I have read the Participant Information Statement and have been given the opportunity to discuss the information and my involvement with the researcher.

3. I understand that being in this study is completely voluntary – I am not under any obligation to consent.

4. I understand that my involvement is strictly confidential. I understand that any research data gathered from the results of my study may be published however no information about me will be used in any way that is identifiable.

5. I understand that I can withdraw from the study at any time without my withdrawal from the study impacting on my medical treatment.

6. I understand that I can withdraw from the study at any time, without affecting my relationship with the researcher or the University of Sydney now or in the future.

7. I understand that I can stop the interview at any time if I do not wish to continue. Should you request it, the audio recording will be erased and the information provided will not be included in the study.

8. I consent to my general practitioner (GP) being contacted by research staff if necessary.

General Practitioners Name: ___________________________________

General Practice Clinic: ___________________________________

Telephone Number: ___________________________________

9. I consent to:

- Audio-recording YES NO
- Receiving Feedback YES NO

If you answered YES to the “Receiving Feedback” question, please provide your details ie. Mailing address or email address.

<u>Feedback Option</u>

Address: ____

____

Email: ____

....

Signature

....

Please PRINT name

....

Date
